# Natural Transformation of *Helicobacter pylori* Involves the Integration of Short DNA Fragments Interrupted by Gaps of Variable Size

**DOI:** 10.1371/journal.ppat.1000337

**Published:** 2009-03-13

**Authors:** Edward A. Lin, Xue-Song Zhang, Steven M. Levine, Steven R. Gill, Daniel Falush, Martin J. Blaser

**Affiliations:** 1 Department of Medicine, New York University School of Medicine, New York, New York, United States of America; 2 Department of Oral Biology, University at Buffalo School of Dental Medicine, Buffalo, New York, United States of America; 3 Mathematical Genetics Group, University of Oxford, Oxford, United Kingdom; 4 Department of Microbiology, New York University School of Medicine, New York, New York, United States of America; 5 VA Medical Center, New York, New York, United States of America; University of Illinois, United States of America

## Abstract

*Helicobacter pylori* are gram-negative bacteria notable for their high level of genetic diversity and plasticity, features that may play a key role in the organism's ability to colonize the human stomach. Homeologous natural transformation, a key contributor to genomic diversification, has been well-described for *H. pylori*. To examine the mechanisms involved, we performed restriction analysis and sequencing of recombination products to characterize the length, fragmentation, and position of DNA imported via natural transformation. Our analysis revealed DNA imports of small size (1,300 bp, 95% confidence limits 950–1850 bp) with instances of substantial asymmetry in relation to selectable antibiotic-resistance markers. We also observed clustering of imported DNA endpoints, suggesting a possible role for restriction endonucleases in limiting recombination length. Additionally, we observed gaps in integrated DNA and found evidence suggesting that these gaps are the result of two or more separate strand invasions. Taken together, these observations support a system of highly efficient short-fragment recombination involving multiple recombination events within a single locus.

## Author Summary

*Helicobacter pylori* are gram-negative bacteria that have been implicated in human diseases after decades of persistence in the stomach. Known for its high level of genetic diversity, *H. pylori* is competent to undergo natural transformation, a process in which donor DNA is integrated into the recipient chromosome. To examine the mechanisms involved, we analyzed the DNA imported via natural transformation in an experimental model system. We found variation in the average length of imported DNA fragments, with asymmetry with respect to a selectable marker. We also found evidence that strain-specific restriction endonucleases may limit recombination length. Additionally, we observed gaps in the integrated DNA and provide evidence that these gaps are the result of separate strand invasions. Together, our observations support a highly efficient system of short-fragment recombination involving multiple recombination events within a small region of the chromosome. This helps explain how bacteria are able to employ genetic recombination to efficiently generate and maintain genomic diversification within the population—a feature that helps *H. pylori* persistently colonize the harsh environment of the human stomach.

## Introduction

Many of the bacterial species that are long-term colonizers of human niches are characterized by high levels of genetic diversity [1]. The gram-negative bacteria *Helicobacter pylori*, which can induce chronic gastritis, peptic ulceration and gastric malignancies after decades of persistence in the human stomach, displays an exceptional level of genomic diversity [2]. Multiple genetically distinguishable isolates have been observed in a single individual, and it has been postulated that genetic diversity and plasticity greatly facilitate adaptation within a host as well as transmission to future hosts [3]–[5]. The finding of highly efficient *in vitro* recombination, along with evidence of genetic shuffling and a panmictic population structure, suggest that horizontal genetic exchange amongst diverse strains plays a substantial role in generating this extensive gene pool [6],[7].

Most wild-type strains of *H. pylori* are naturally competent for transformation, which occurs via the actions of proteins encoded by the *comB* locus [8]–[13]. Horizontal transfer of donor chromosomal DNA containing a point mutation conferring a selectable antibiotic resistance phenotype can be observed in the homeologous natural transformation of *H. pylori* [9]. The genome of the transformed strain includes a point mutation flanked by varying lengths of the donor DNA. Better knowledge of the characteristics of homeologous recombination may aid in understanding how *H. pylori* persists through changing environments within the human host, as well as serving as a model for other naturally competent organisms.

Prior studies, which involved mathematical analysis of *H. pylori* genomic sequence data, provide evidence that the median size of imported donor fragments appears small (412 bp) compared with *Streptococcus pneumoniae*, *Neisseria meningitidis*, and *Bacillus subtilis* (2000, 5000, and 10,000 bp, respectively) [14]–[17]. In addition, as little as 5 bp flanking an antibiotic resistance-conferring point mutation and 150 bp flanking an antibiotic cassette are sufficient to allow transformation to antibiotic resistance [8],[18]. In this study, we present a new approach to studying the products of transformation by taking advantage of the naturally-occurring polymorphisms between two sequenced *H. pylori* strains. We employed restriction analysis and direct sequencing of recombination products to define the length of an imported sequence flanking a point mutation conferring antibiotic-resistance. Using this approach, we were also able to characterize the fragmentation and configuration of this imported DNA. Additionally, we examined whether our findings could be explained by self-hybridization, methylation, or restriction modification of the recombination substrates. Finally, we were able to confirm and compare the transformation characteristics involving more than one genomic locus.

## Results

### Transformation of strain J99 with 26695 Str^R^ chromosomal DNA

**Figure 1:**
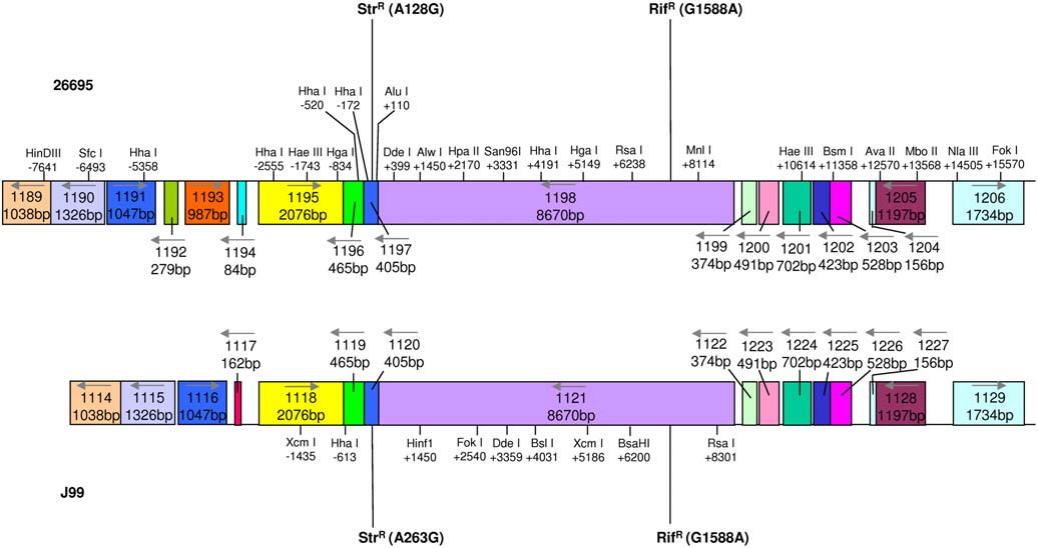
Schematic of the region flanking the *rpsL* Str^R^ mutation in *H. pylori* strains 26695 and J99. The map includes restriction sites unique to one or the other strain and the distance (bp) upstream (−) or downstream (+) from the Str^R^ site (A128G in HP1197 of strain 26695, and A263G in JHP1120 of strain J99). Four-digit numbers reflect ORF designations in the annotated genome sequences, and the numbers below these reflect ORF length (bp). Colors indicate ORF homology between the two strains. The grey arrows indicate the direction of transcription. “Downstream” and “upstream” refer to the direction of replication from the origin.

Under the standardized conditions employed [8], chromosomal DNA from 26695 Str^R^ cells transformed J99 wild-type cells to Str^R^ with a mean frequency of 4.56×10^−5^ transformants/CFU/µg DNA (Table 1). These data were consistent with those from previous experiments ([8], data not shown). This frequency of homologous transformation was approximately 10-fold higher than for homeologous transformation with J99 donor chromosomal DNA [8]. Natural transformation resulted in the incorporation of a point mutation (A128G in codon 43 of *rpsL* in strain 26695 donor DNA) into the recipient strain J99 genome. Our first goal was to determine the length of DNA flanking the point mutation (conferring streptomycin resistance) that was incorporated into the recipient genome along with the mutation. Since there is a ∼6% genetic variation within conserved ORFs between the two strains [19], the flanking DNA includes numerous single nucleotide polymorphisms (SNPs), that can differentiate between the two strains as the source of a mosaic DNA. For the 8790 bp flanking the Str^R^ site, there were 627 SNPs (7.1% polymorphism) between the strains (mean separation 20.3±19.3 bp). Those SNPs that were within one of the pre-designated restriction sites (Figure 1) were identified.

**Table 1:** Comparison of transformation frequency and flanking DNA length in homeologous transformation of *H. pylori* strains 26695 and J99.

Recipient Strain	J99	26695	J99	26695
Donor Strain^a^	26695 Str^R^	J99 Str^R^	26695 Cm^R^ (HPXZ135)	J99 Cm^R^ (HPXZ136)
Transformation frequency (transformants/cfu/ug DNA)	4.56×10^−5^	1.67×10^−6^	5.6×10^−6^	2.98×10^−6^
Mean (±SD) length of flanking DNA upstream of mutation/cassette (bp)^b^	683±445*	1472±965^c^ *	1695±795	1771±936
Mean (±SD) length of flanking DNA downstream of mutation/cassette (bp)^b^	1375±1494	1342±1378^c^	1392±1157	1623±959
Mean (±SD) length of imported DNA (bp)^b^	2058±1494	2814±1542^c^	3098±946	3393±1013

a Source of chromosomal DNA encoding antibiotic resistance used to transform recipient host strain.

b Mean (±SD) for ten independent transformants.

c For regions in which the inserted DNA was discontinuous, only donor DNA was used to determine the length of flanking DNA.

*: p<0.05.

In each independent transformant, we sought to determine the presence or absence of such restriction sites at various distances from the Str^R^ mutation. To achieve this, PCR products were amplified using primers flanking the restriction site (Table S1), and the resulting amplicons were treated with the appropriate restriction enzyme. At distances 100 bp and 399 bp downstream of the Str^R^ (A128G) mutation, all transformants from 10 independent transformations of strain J99 contained restriction sites unique to the donor strain, 26695 (“downstream” or “upstream,” refers to the direction of replication from the origin). However, at 811 bp, 3331 bp, 4668 bp, and 5149 bp downstream of A128G, the number of independent transformants containing the 26695-unique sites fell to 5, 2, 1, and 0, respectively. At a distance of 172 bp upstream of A128G, 9 of the 10 independent transformants analyzed contained the donor 26695-unique sites. That frequency fell to 5, 4 and 0 transformants at increasing distances upstream of A128G (520, 834, and 1350 bp, respectively). No two of the 10 studied transformations yielded identical results, confirming that these transformants were truly independent. The presence or absence of a restriction site was unambiguous within any of the independent transformants, making it unlikely that mixed clones were present.

**Figure 2:**
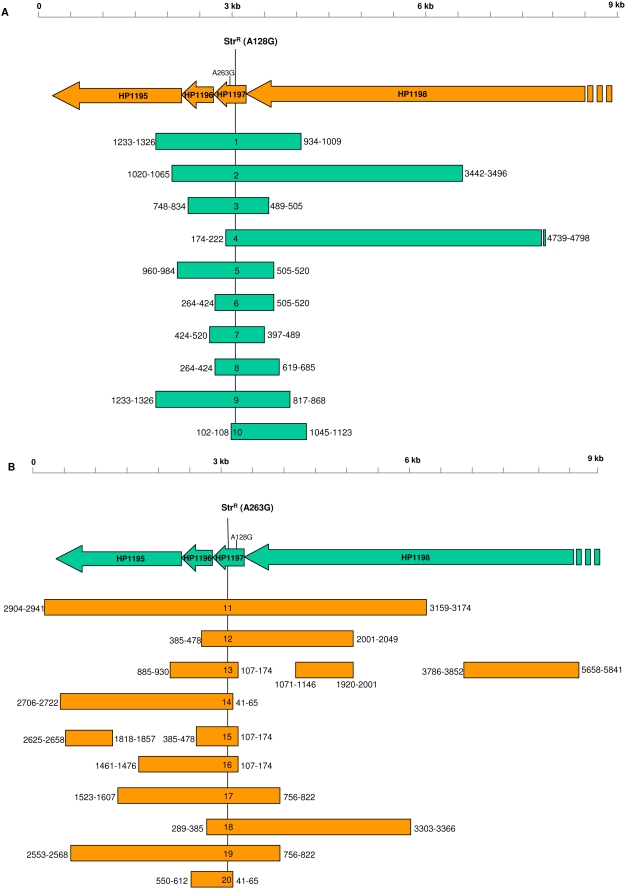
Length of imported DNA flanking the point mutation conferring Str^R^ following transformation with chromosomal DNA from a donor Str^R^ *H. pylori* strain. The values on either side of the bars indicate the distance (bp) from the Str^R^ mutation to two single nucleotide polymorphisms (SNPs), reflecting differential susceptibility to restriction endonucleases that define the recombination endpoints. The endpoint of each DNA segment was designated as the position halfway between the two SNPs. The recombination endpoints were determined by sequencing ∼1 kb near ends indicated by restriction analysis of PCR amplicons. (A) Donor DNA from Str^R^ strain 26695 → Recipient J99 cells. The point mutation in 26695 gene HP1197 conferring streptomycin resistance (A128G) was present in all ten independent transformants; an alternate streptomycin resistance mutation (A263G) present in naturally Str^R^ J99 was not observed. (B) Donor DNA from Str^R^ strain J99 → Recipient 26695 cells. The point mutation in JHP1121 conferring streptomycin resistance (A263G) was present in all ten independent transformants; an alternate streptomycin resistance mutation (A128G) present in naturally Str^R^ 26695 was not observed. For transformants 11,12,14,15,16, and 17, the 8517 bp region (from 2278 bp upstream to 6239 bp downstream) that flanks the Str^R^ mutation was subjected to sequence analysis to completion.

For each independent transformant, the restriction sites furthest upstream and downstream from Str^R^ that were still unique to 26695 (donor) were identified as proximal to the crossover endpoint. To discriminate the crossover endpoints with greater precision, PCR products were amplified using primers flanking these restriction sites, and sequences of the amplicons were determined. For each, the sequence data revealed a region that was bounded by two SNPs—one unique to 26695 and the other, to J99. To calculate flanking DNA length, the crossover endpoint was defined as the middle of this region. For the 10 independent transformants, the lengths of flanking DNA ranged from 915–4967 bp (Table 1). In total, we observed the mean integration of 2058±1494 bp donor DNA into the recipient chromosome. Despite substantial variation among flanking DNA lengths, two independent transformants were found to contain crossover endpoints within a single 15 bp region (transformants 5 and 6 showed crossovers 505–520 bp downstream of Str^R^) (Figure 2A).

Further analysis of transformant 4 revealed a 13 bp region (4742–4754 bp downstream of the Str^R^ mutation) that can be characterized as a gap in integration. This region contained a SNP unique to J99 (recipient) that was flanked by 2 SNPs unique to 26695 (donor). This result was observed in sequences obtained with both the forward and reverse primers.

The integration of DNA was asymmetric with respect to Str^R^, favoring the downstream side at a ratio of about 2∶1. Several transformants had integrated flanking DNA with extreme asymmetry, such as transformant 4, which was characterized by a downstream∶upstream ratio of 24∶1.

### Transformation of strain 26695 with J99 Str^R^ chromosomal DNA

To examine the hypothesis that flanking DNA asymmetry is influenced by a region of non-homology between 26695 and J99 occurring ∼3 kb upstream of A128G (Figure 1), we repeated the transformation experiment but reversed the roles of the strains. Chromosomal DNA from J99 Str^R^ cells transformed 26695 wild-type cells to Str^R^ with an average efficiency of 1.67×10^−6^ transformants /CFU/µg DNA (Table 1). Natural transformation resulted in the incorporation of a point mutation (A263G in codon 88 of *rpsL* in strain J99) into the recipient strain 26695 genome. Surprisingly, at a distance as small as 110 bp downstream of Str^R^, 5 of 10 transformants contained a restriction site unique to *recipient* strain 26695, indicating that the contiguous downstream crossover point was very close to the A263G transforming point mutation. Sequence data confirmed this extreme asymmetry with some of the crossover endpoints located only 53 bp downstream of A263G (Figure 2B). Several transformants had very high upstream∶downstream flanking DNA ratios; for example, transformant 14 displayed a ratio of 51∶1.

An average of 2814±1542 bp of flanking DNA was integrated along with the point mutation, with flanking DNA lengths ranging from 634 to 6089 bp. The sequence data for transformants 13 and 15 revealed large gaps of integration, in which the flanking donor DNA is interrupted by significant regions (up to 1658 bp) of recipient DNA (Figure 2B). To examine the flanking DNA for additional gaps, DNA from six independent transformants (11,12,14,15,16, and 17) was completely sequenced within a ∼8.5 kb region from 2278 bp upstream to 6239 bp downstream of the Str^R^ mutation. The sequence data confirmed the identity of the polymorphisms that had been deduced by the previous restriction site analysis (Figure 2B). No additional gaps of integration were observed. Thus, out of 20 independent transformants, only 3 were found to contain gaps (transformants 4, 13, and 15).

Although several transformants had flanking DNA characterized by extreme asymmetry, across the 10 independent transformants, the mean sizes of the flanking DNA fragments upstream and downstream from A263G were similar (1472 bp±965 upstream and 1341 bp±1378 downstream). The flanking DNA of transformant 11 extends upstream by nearly 3 kb and borders the region of non-homology between the two strains. Although no significant difference in total flanking DNA length was found between the two strains, recipient strain 26695 contained significantly more upstream flanking DNA than J99 (Table 1). This is not unexpected since the region of non-homology between the two strains lies upstream in relation to the Str^R^ mutation.

### Statistical analysis of import size

To estimate the mean size of imports caused by transformation of chromosomal *H. pylori* DNA, we performed statistical analysis that takes into account both the uncertainty in the position of the recombination endpoints due to the stretches of sequence identity between J99 and 26695 and the fact that longer imports are more likely to appear in the dataset because they are more likely to include the Str^R^ mutation used to select for strains that have undergone recombination (see Methods). The analysis does not take into account the gaps in integration described above, which are difficult to model statistically. Instead, in a first analysis, we only took into account the stretch of contiguous imported DNA immediately flanking the Str^R^ mutation. This analysis gave a mean import size of 1100 bp (95% confidence limits 800–1600 bp). A second analysis, treating the whole imported stretch as a single unit, and ignoring the effect on ascertainment of the gaps in integration, yields an estimate for the mean size of the entire region involved in the import of 1300 bp (95% confidence limits 950–1850 bp).

### Chromosomal markers of enhanced recombination

That no two of the transformants yielded identical results, confirms the independence of each event. However, despite significant variation among flanking DNA lengths, two transformants were found to contain crossover endpoints within a single 15 bp region (transformants 5 and 6 downstream of Str^R^). Two other transformants (14 and 20) shared an endpoint within 24 bp. Still other transformants (17 and 20) contained crossover endpoints within a single 66 bp region downstream of Str^R^. Indeed, the endpoints of many transformants were found to be clustered within short regions—transformants 13, 15, and 16 were within a 67 bp region; transformants 14 and 15 were within a 97 bp region; transformants 12 and 15 were within a 93 bp region; transformants 1 and 9 were within a 93 bp region; transformants 15 and 19 were within a 105 bp region; transformants 2 and 5 were within a 105 bp region; and transformants 3 and 7 were within a 108 bp region. Of 48 crossover points in the 20 independent transformants studied in the two experimental protocols, 20 (41.7%) recurred within short regions (<110 bp). These short regions did not coincide with regions of putative self-hybridization, as determined by MFold [20].

*H. pylori* strains contain numerous functional methyltransferases that protect endogenous DNA from endonuclease digestion by methylating specific recognition sequences [21]. Methylated genomic DNA may interrupt donor DNA integration during recombination, generating multiple crossover endpoints within a single short region. To explore the hypothesis that genomic DNA methylation patterns play a role in determining crossover endpoints during homeologous transformation, the locations of the methyltransferase recognition sequences in both 26695 and J99 were mapped alongside the crossover endpoints in the 20 independent transformants. This comparison did not yield any significant correlations (data not shown), suggesting that methylation of neither the recipient chromosome nor the donor DNA significantly interrupts the integration of donor DNA.

**Figure 3:**
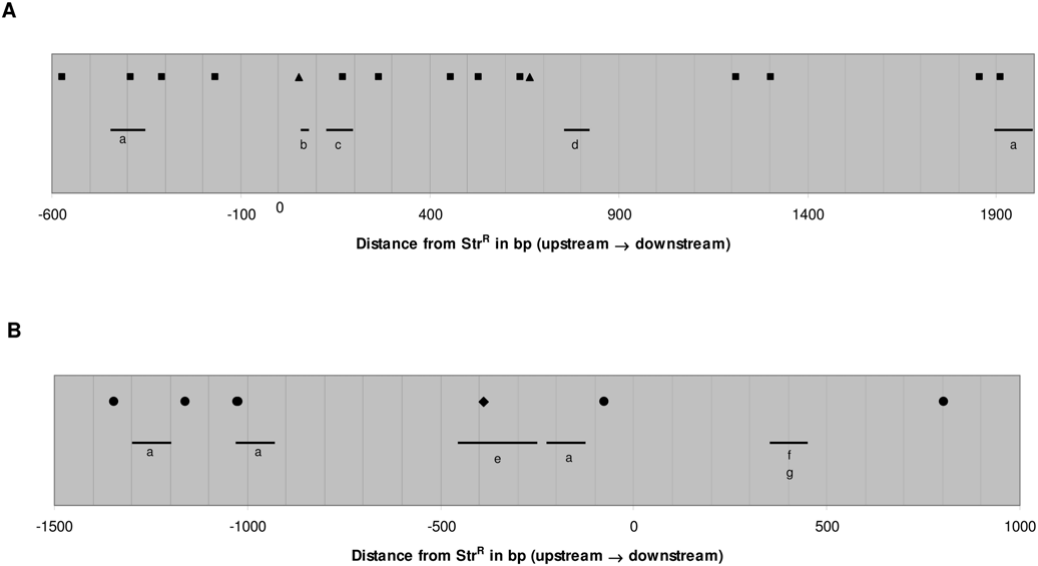
Alignment of methylation sites and regions containing multiple crossover endpoints in *H. pylori* DNA. Each panel is a representation of ∼2500 bp flanking a Str^R^ site in strain J99 or 26695. Each horizontal bar represents a region that contains at least two recombination endpoints: a, two endpoints within 100 bp; b, two endpoints within 24 bp; c, three endpoints within 68 bp; d, two endpoints within 70 bp; e, two endpoints within 200 bp; f, two endpoints within 15 bp; g, four endpoints within 100 bp. (A) Map of sites in chromosomal DNA from strain J99 recognized by active 26695 restriction endonucleases for which the corresponding methylase is absent in J99. *HpyAII* recognition sites (GAAGA) are designated by filled squares (■). *HpyAV* recognition sites (CCTTC) are designated by filled triangles (▲). (B) Map of sites in 26695 chromosomal DNA recognized by active J99 restriction endonucleases for which the corresponding methylase is absent in 26695. *Hpy99IV* recognition sites (CCNNGG) are designated by filled circles (●). *Hpy99I* recognition sites (CGWCG) are designated by filled diamonds (◆).

*H. pylori* strains 26695 and J99 each encode four different active restriction-modification (R-M) systems [22]. Each system consists of an active restriction endonuclease (RE) and a methytransferase, both recognizing the same sequence. Since the R-M systems are not conserved between the two strains, the donor DNA in homeologous transformation is susceptible to cleavage by REs active in the recipient strain. To examine the role of endonuclease restriction of donor DNA in homeologous transformation, the locations of donor DNA recognition sequences targeted by active recipient strain REs, for which the corresponding methyltransferase is either absent or non-functional in the donor strain, were mapped alongside the crossover endpoints in the 20 transformants (Figure 3). In total, 11 of 23 (48%) crossover endpoints were within regions that overlapped with the specific RE recognition sites. To determine whether this was a chance observation, we performed statistical inference modeling, considering the endpoints of the contiguous imported fragment flanking Str^R^ as being γ times more likely to occur at sites that are susceptible to cleavage due to restriction modification than at sites that are not. This analysis yielded an estimate of γ of 32. However the confidence limits are wide, ranging from 0 to 100, so that based on these 20 events, we have insufficient power to determine whether the restriction sites are significantly overrepresented at import boundaries.

### Transformation of 26695 with PCR products containing one or two antibiotic-resistance alleles

The observation of gaps in integration has been described previously in *Streptococcus pneumoniae*, and has been attributed to the activity of mismatch repair (MMR) proteins [23]. However, *H. pylori* does not contain homologs to known MMR genes [19],[24], and has no MMR activity [25]. Since the efficiency of natural transformation was on the order of 10^−6^, the predicted frequency of adjacent independent recombination events would be ∼10^−12^. Therefore, we hypothesized that these gaps are the result of DNA repair enzyme activities, rather than adjacent independent recombinations. To examine this hypothesis, we analyzed natural transformation of 26695 cells with a ∼9 kb PCR product (designated P-StrRif) amplified via primers flanking the A128G mutation and a point mutation conferring rifampin-resistance (G1588A in *rpoB*, resulting in D530V). The mutation conferring Rif^R^ is separated from the mutation conferring Str^R^ by ∼7.2 kb (Figure 1). Such transformation yielded Str^R^ transformants and Rif^R^ transformants with a frequency of ∼10^−4^ transformants/CFU/ugDNA, but Str^R^Rif^R^ transformants (containing both mutations) with a frequency of 4.7×10^−6^ transformants/CFU/ug DNA (Table 2). These findings suggest a linkage between the two markers. However, since both markers are co-localized on a single PCR product, it is not clear whether this linkage was the result of a single DNA crossover event or multiple ones. To differentiate between these hypotheses, we also transformed 26695 cells with an equimolar mixture of the ∼9 kb PCR products containing *either* the Str^R^ mutation (designated P-Str) *or* the Rif^R^ mutation (designated P-Rif). Under these conditions, transformation efficiencies were similar to those from the single product (Table 2). The frequency of finding an integrated Rif^R^ mutation adjacent to a given Str^R^ mutation (or visa versa) was ∼1/130. That this frequency is several log_10_ higher than the frequency predicted for two adjacent independent recombinations, suggests that the integration of both markers is mechanistically linked, and that such linkage occurs even if both markers are located on separate DNA molecules.

**Table 2:** Efficiency of natural transformation of *H. pylori* 26695 cells involving two selectable markers.

Rifampin	Streptomycin^a^	P-StrRif^b^	P-Str+P-Rif	No PCR Product (Control)
+	−	3.0×10^−4^	2.2×10^−4^	<10^−9^
−	+	4.3×10^−4^	4.3×10^−4^	<10^−9^
+	+	4.7×10^−6^	4.4×10^−6^	<10^−9^

a BS plates contained streptomycin (20 µg/ml) and/or rifampin (87.5 µg/ml).

b For each transformation, 100 ng of each donor 9 kb PCR product was used. Values are calculated as transformants/cfu/µg DNA, based on comparisons of the non-selective and the selective media.

To further explore this linkage, we repeated the experiment using J99 cells and a donor ∼9 kb PCR product derived from a 26695 Str^R^ Rif^R^ template. Using restriction analysis (as described above), transformant chromosomal DNA was analyzed for the presence of a SNP unique to J99 (recipient) that was approximately equidistant from the Str^R^ and Rif^R^ mutations. Such analysis confirmed the presence of the SNP in 6 of 6 transformants, suggesting that within the transformant DNA, a gap in integration exists between the two antibiotic resistance mutations. The presence of such a gap is consistent with a mechanism involving multiple crossover events. This analysis was also repeated after reversing the strains. 26695 cells were transformed with a donor ∼9 kb PCR product derived from a J99 Str^R^Rif^R^ template, yielding Str^R^Rif^R^ transformants which were analyzed for the same SNP. This analysis found that four of the six independent transformants contained the SNP unique to 26695 (recipient), but two transformants had the donor SNP. While this finding does not rule out the presence of a gap in integration between the Str^R^ and Rif^R^ mutations in these two transformants, it suggests that multiple mechanisms may be involved in the generation of these genetic products during homeologous transformation.

### Transformation involving an alternate locus

**Figure 4:**
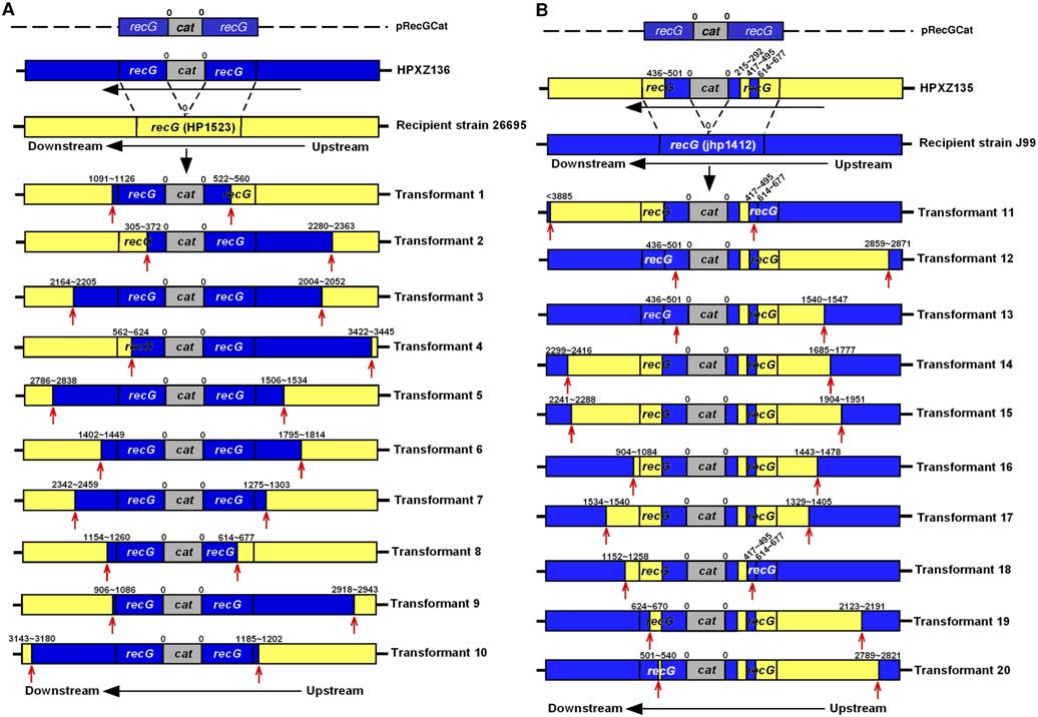
Length of imported DNA flanking a *cat* cassette following transformation with chromosomal DNA from donor *H. pylori* strains. The values above the bars indicate the distance (bp) from the *cat* cassette to the recombination endpoints (red arrows), as determined by sequence analysis. The numbers reflect the distance from the nearest cassette boundary; the range reflects the locations of informative SNPs. Sequences corresponding to J99 are colored blue; those corresponding to 26695 are colored yellow. (A) J99-derived donor HPXZ136 transforming 26695 cells. (B) 26695-derived donor HPXZ135 transforming J99 cells.

The *rpsL* gene encodes an abundant ribosomal binding protein, and is located within a highly transcribed region of the genome. To evaluate the products of natural transformation in other less-highly transcribed genomic regions, we studied the region of *recG*. Previous data involving microarrays have shown that the transcription rate of *recG* is 15 to 87-fold less than that of *rpsL* [26]. For our studies, we used the pRecGCat plasmid, as described previously [27]. pRecGCat contains a sequence corresponding to *recG* of strain J99 that is interrupted by a chloramphenicol acetyl transferase (*cat*) cassette conferring chloramphenicol resistance (Cm^R^). In initial studies, we transformed pRecGCat into wild-type J99 cells to generate strain HPXZ136. Chromosomal DNA from this strain then was used to transform 26695 cells; 10 independent transformants were isolated, as in the prior experiments. The frequency of transformation was 3.0×10^−6^±2.5×10^−6^ transformants/CFU/µg DNA, which is ∼10-fold less than for the transformations involving the Str^R^ point mutation. The transformation frequency for the control experiment (J99 *recG* cat→J99) was similar (6.3×10^−6^±3.1×10^−6^ transformants/CFU/µg DNA), indicating no large restriction barrier for this transformation into 26695. As in the prior experiments (see Materials and Methods), we performed sequence analysis on 10 independent transformants, yielding the locations of the upstream and downstream crossover endpoints (Figure 4A). In total, in addition to the selectable 884 bp *cat* cassette, the mean flanking insert size was 3393±1013 bp (range 1650–4355 bp). The average lengths of the upstream (1771±936 bp) and downstream (1623±959 bp) inserted sequences were similar, although the actual fragment lengths varied considerably. This is >1 kb more than the mean length of integrated DNA observed for the Str^R^ mutation. No gaps in integration were observed within any of the 10 transformants, nor was there clustering of crossover endpoints.

Strain 26695 contains two active restriction endonucleases (*HpyAII* and *HpyAV*) for which J99 (and thus HPXZ136) lacks the cognate methylases. Within the range of the cross-overs, there are 19 upstream and 12 downstream sites on the J99 chromosome that would be cleaved by these 26695 REs. In total, 6 (30%) of the 20 endpoints overlap with the specific restriction sites, a finding significantly greater than chance (p = 0.046).

In a second experiment involving the *recG* locus, we used a more complex transformation strategy (Figure 4B). We transformed pRecGCat into wild-type 26695 cells to create strain HPXZ135. Sequence analysis of HPXZ135 revealed a ∼200 bp segment of 26695 sequence interrupting the J99 sequence immediately upstream of the *cat* cassette (Figure 4B). This ∼200 segment was designated as the “donor gap.” We then used chromosomal DNA from HPXZ135 (containing the “donor gap”) to transform wild-type J99 recipient cells. 10 independent transformants were isolated, as in previous experiments. This transformation yielded colonies at a mean efficiency of 5.60×10^−6^±6.93×10^−6^ transformants/CFU/µg DNA, similar to the results when HPXZ136 was used. Sequence analysis performed on the 10 independent transformants identified the locations of upstream and downstream crossover endpoints (Figure 4B). In total, we observed a mean integration of 3098±946 bp donor DNA into the recipient chromosome, with similar upstream (1695±795 bp) and downstream (1392±1157 bp) sequence lengths. In each transformant, the architecture of the donor DNA was preserved, with the *cat* cassette and the “donor gap” present in all transformants (Figure 4B). The transformation fragment variation reflected the location of the crossovers involving the flanking 26695 sequences. As in the prior analysis (Figure 4A), we did not observe any clustering of endpoints.

We mapped the sites of known active J99 restriction endonucleases (*Hpy99I*, *Hpy99II*, and *Hpy99IV*) whose corresponding methylases are absent in 26695 and thus, HPXZ135. We found relatively few RE sites flanking the *cat* cassette, with only 4 sites within 4 kb downstream of the cassette (708, 2097, 3019, and 3020 bp from the cassette) and none upstream. With so few informative markers, we found no relationship between crossover points and restriction sites.

## Discussion

In *S. pneumoniae*, *N. meningitidis* and *B. subtilis*, transformation yields imported DNA lengths of about 2 kb, 5 kb and 10 kb, respectively [15]–[17]. It might have been anticipated that transformation of *H. pylori* would result in imported DNA lengths at the upper end of this range since *H. pylori* lacks homologs to DNA recombination/repair proteins [28], which have been implicated in limiting the length of heteroduplex formation during recombination in both prokaryotes and eukaryotes [29]. However, natural transformation of *H. pylori* yields insertion lengths near the smaller portion of this range, with a mean integration of ∼2400 bp of donor chromosomal DNA into the recipient chromosome (Table 1). After statistical treatment of the data, the mean import size was found to be ∼1300 bp, which is consistent with previous reports of small import lengths within naturally occurring mosaics of *H. pylori*, as well as efficient *in vitro* transformation with small donor DNA fragments [8],[14]. Although it has been suggested that digestion of naked DNA within the external environment may play a role in reducing the length of imported fragments, a significantly greater mean import length was not observed despite limiting the donor substrate to freshly purified chromosomal DNA extract. While excessive mechanical shearing of the donor DNA cannot be excluded, it is generally prevented with standard techniques and would not explain the astochastic distribution of crossover endpoints [30].

In other naturally competent organisms, such as *N. gonorrhoeae*, *S. pneumoniae*, and *B. subtilis*, DNA is imported as a single strand that is resistant to cleavage by endonucleases [31]–[33]. However, there is evidence to suggest that natural transformation in *H. pylori* may be different. Natural competence in *H. pylori* is associated with proteins at the *comB* locus—a feature not found in other naturally competent bacteria [11]–[13]. Transformation of *H. pylori* involving double-stranded donor DNA results in ∼1000-fold higher transformation efficiency than transformation with a single-stranded substrate [8]. Furthermore, evidence of a restriction barrier has been found in *H. pylori*; for example, strain-specific methylation of donor DNA transforms *H. pylori* more efficiently than unmethylated DNA that is susceptible to restriction [34]–[36]. If substrate donor DNA exists in the cytoplasm as a double-strand, the action of native strain-specific *H. pylori* restriction endonucleases would be predicted to curtail import size. In our data, we observed an overrepresentation of crossover endpoints at susceptible restriction sites, which would be the expected consequence of donor substrate DNA restriction. It would be interesting to compare our findings with those in strains that do not harbor R-M systems.

The finding of imports with extreme asymmetry with respect to the selective Str^R^ mutation is consistent with the previous observation that *H. pylori* is successfully transformed by asymmetric donor DNA fragments with minimal homology on one flank and is suggestive of a highly efficient mechanism for recombination [8]. This finding is also consistent with the observation that flanking DNA in one transformant actually abuts the region of non-homology between strains 26695 and J99. This is unsurprising in an organism that lacks MMR enzymes, since such enzymes would be expected to play a role in the early termination of recombination at polymorphic sites.

The observation of gaps of integration has been previously described for *S. pneumoniae* [23]. In bacterial transformation, initiation of recombination involves the invasion of a single-stranded segment of donor DNA into the recipient chromosome. The finding of gaps of integration has been previously attributed to events that occur following donor strand invasion, such as repair excision of mismatched bases, resulting in the conversion of genetically heterozygous sites to homozygosity [23],[37]. However, since *H. pylori* lacks a full complement of mismatch repair enzymes, we hypothesized that these gaps may be due to events occurring before or during donor strand invasion. For example, the initiation of two separate but adjacent strand invasion events would result in two neighboring regions of donor genotype separated by a gap.

However, the coincidence of two or more independent strand invasion events within ∼3 kb (see Figure 2B) would be unexpected given our sample size, unless the events were mechanistically linked. One example of linkage would involve the origination of both invading strands from a contiguous DNA molecule. However, we found that the simultaneous genomic integration of two genetic markers present on two separate DNA molecules occurred with the same frequency as the integration of markers coexisting on the same molecule (Table 2). While multiple hypotheses exist to account for such linkage, including local concentration effects due to enzyme recruitment, the uptake of large quantities of donor DNA by the cell, and increased susceptibility of certain genomic regions to recombination, these findings suggest that any explanation for the mechanism of linkage between the two markers must include an account for adjacent strand invasions involving at least two discrete DNA molecules. This finding is certainly consistent with a donor substrate that has been cleaved by R-M system enzymes prior to strand invasion of the chromosome. However, the Str^R^ and Rif^R^ mutations are located in highly transcribed essential genes, for which the measured recombination rate may differ from that in other parts of the genome.

It might be suggested, that the observation of relatively large gaps within a 9 kb region and the finding of much smaller gaps within a ∼3 kb region (Figure 2B), are the result of two different processes. While the incidence of gaps in the 9 kb region is significantly lower than for the 3 kb region (an incidence of ∼1/130 for the 9 kb region versus 3/20 for the 3 kb region), that may not be unexpected since two simultaneous strand invasions that are far apart may occur much less frequently than strand invasions that are closer together. Furthermore, the finding that the incidence of gaps varies at 9 kb versus 3 kb suggests that location and proximity of markers does indeed play a role, and that the simultaneous integration of both markers is not simply the result of two coincidental but independent events. The finding of significant gaps within the 9 kb region strongly suggests that the distance between the Str^R^ and Rif^R^ markers (∼7.2 kb) may be too long for a single co-transformation event. Thus, each doubly marked fragment only transforms at a single site, but not at both. These observations set an upper boundary (of 7.2 kb) for the amount of DNA that can be contiguously integrated from a single site, but may reflect the restriction and methylation differences between the donor and recipient strains.

To examine whether the same recombination characteristics are present for non-essential genes, we transformed wild-type 26695 cells with chromosomal DNA from strain HPXZ136, which contains a *cat* cassette within the *recG* gene. Interestingly, the frequencies of transformation between *recG* and *rpsL* were similar (Table 1). Given that the transcription rate of *recG* is up to 87-fold lower than that of *rpsL* [26], this suggests that differences in transcription rate may not have a major effect on transformation efficiency. Genomic sequencing of the resulting transformants showed no evidence of gaps in integration or clustering of endpoints, as had been found flanking the Str^R^ mutation in *rpsL*. However, there was a significant overrepresentation of endpoints that overlapped with sites in which the incoming J99 DNA would be susceptible to 26695-specific restriction. As a further test, we performed a transformation involving a more complex genomic architecture from strain 26695 into J99. The frequency of transformation was almost the same as when the simpler architecture was used, indicating no substantial differences whether or not a “donor gap” in the identity of the sequence was introduced. The lack of endpoint clustering was consistent with the very small number of J99-specific restriction sites on the incoming 26695-based donor DNA. Of note, the distribution of flanking DNA was relatively symmetric with respect to the *cat* cassette, a finding that contrasts with the asymmetric distribution of DNA flanking the Str^R^ mutation. This may be due to the loss of homology ∼3 kb upstream of the Str^R^ mutation in *rpsL*, a feature not found in *recG*.

One potential limitation of our technique is the lack of fully-quantitative standardization of cell density prior to the introduction of donor DNA. However our method of standardizing cell density has yielded highly consistent transformation efficiencies over time, and the transformation frequencies we have observed are consistent with previous experiments [8].

In conclusion, we have presented a new approach to studying the products of transformation that has yielded evidence of highly efficient short-fragment recombination involving multiple recombination events within a single locus. These events likely involve distinct genetic markers residing on discrete DNA molecules, as is the case following processing by cell-specific restriction endonucleases. While our findings may also be partially explained by the lack of a mismatch repair pathway in *H. pylori*, it would be worthwhile to consider the role of other proteins that regulate recombination, such as *MutS2* [38]. It also would be of interest to consider the role of various type I and type III R-M systems that are present in *H. pylori*. By phylogeography, 26695 is an hpEurope strain while J99 is of the hpAfrica1 lineage, which suggests that they are not closely related despite commonalities within certain loci (eg. *cag* and *vacA*) [39]. It may be interesting to explore whether genetic exchange between more-closely related strains, with fewer R-M system barriers, yields different results. Within this context, we have uncovered novel and interesting insights into how recombination occurs around a selectable genetic marker. This will help us to further understand how bacteria are able to employ genetic recombination to efficiently generate and maintain genomic diversification within the population—a feature that allows *H. pylori* to persistently colonize the human stomach [28].

## Materials and Methods

### Strains and growth conditions

The *H. pylori* isolates used in this study were wild-type strains 26695 and J99, for which the complete genomic sequences have been solved [19],[24]. *H. pylori* cells were grown at 37°C in 5% CO_2_ for 48 h on Trypticase soy agar (TSA) with 5% sheep blood (BBL Microbiology Systems, Cockeysville, MD) or *Brucella*-serum (BS;BBL) agar with 10% newborn calf serum (Serologicals Corporation, Norcross, GA) plates. Spontaneously streptomycin-resistant (Str^R^) mutants of these strains were selected by plating approximately 10^10^ cells on TSA medium containing streptomycin (10 µg/ml). This procedure yielded *H. pylori* strains that had single point mutations conferring streptomycin resistance in *rpsL*; for 26695, the mutation was A128G (K43R), and for J99, the mutation was A263G (K88R) [9],[40]. For the transformation experiments involving donor DNA, independent transformants were selected on BS plates with streptomycin (20 µg/ml) added. Rifampin-resistant (Rif^R^) *H. pylori* strains were obtained by natural transformation of 26695 cells with PCR products amplified using primers containing an A1589T (D530V) point mutation in *rpoB* that confers rifampin resistance [41]. Each independent transformant was harvested from a single colony growing on a separate plate, to ensure that each transformant represented an entirely independent genetic clone. A chlorampheniol-resistant (Cm^R^) J99 strain, HPXZ136, was generated by transforming wild-type J99 with plasmid pRecGCat which is based on J99 *recG* [27], and then selecting one single colony from a BS plate containing chloramphenicol (10 µg/ml). The *cat* cassette insertion site in the HPXZ136 genome and its flanking region was confirmed by sequencing. A second transforming DNA was prepared by using pRecGCat (with J99 *recG*) to transform strain 26695, with the same selection and confirmation as above, creating HPXZ135. This strain has a 200 bp gap of 26695 sequence within the J99 sequence as a result of the pRecGCat transformation.

### DNA techniques

*H. pylori* chromosomal DNA was prepared from cells of each independent transformant after 48 h of growth on TSA plates, as described [30]. PCR was performed on the isolated DNA in a reaction volume of 100 µl containing 1U of *Taq* (Qiagen, Valencia, CA) as per manufacturer's protocol. Each pair of primers was selected to exactly match the chromosomal sequences in both J99 and 26695 (Table S1), and flanked a restriction site unique to that segment for either 26695 or J99 (Figure 1). Restriction enzyme (RE) digestions were performed in a reaction volume of 20 µl containing 2U of the appropriate RE in its buffer at 37°C for 12 h. All PCR products were electrophoresed at 120V for 60 min through 2% agarose gels with 0.7 µg/mL ethidium bromide. The desired band was excised from the gel, purified (Qiagen, Valencia, CA), and stored at −20°C until used in transformation studies.

### Natural transformation

The *H. pylori* cells to be transformed were grown on trypticase soy agar (TSA) plates with 5% sheep blood (BBL) agar for 48 h, harvested into 1 ml of phosphate-buffered saline (PBS) pH 7.4, centrifuged at 850 *g* for 5 min, and the pellet resuspended in 150 µl of PBS. Each transformation mixture, consisting of 25 µl of recipient cells (∼10^8^ cells) and 15 µl (at 2 ng/µl) of donor cell chromosomal DNA, was spotted onto a TSA plate and the plates were incubated for 24 h at 37°C in a 5% CO_2_ atmosphere. The transformation mixture then was harvested into 1 ml of PBS, and 100-µl aliquots of appropriate serial dilutions inoculated to both TSA (non-selective) and antibiotic (selective) plates with 20 µg/ml streptomycin, 87.5 µg/ml rifampin, or both, and incubated for 5 days at 37°C in a 5% CO_2_ atmosphere. The number of colonies of transformants and total viable cells were counted and the transformation frequency was calculated as the number of streptomycin-resistant colonies per microgram of DNA per recipient CFU. Transformation using the *cat* cassette was performed similarly, except that 15 µl (containing 100 ng) of donor chromosomal DNA was used. Cm^R^ transformants were selected on BS agar plates with 10 µg/ml chloramphenicol.

### Mapping the transforming fragment

For each recipient strain, a single aliquot of cells harvested from a fully confluent plate, was divided into 25 ul portions, which were each placed in the center of a separate TSA plate. Donor DNA was then introduced to each plate and the plates were incubated overnight. The resulting transformed cells from each plate were then harvested, diluted, and inoculated onto an antibiotic plate. One colony from each antibiotic plate was selected with a sterile pipette, inoculated onto a TSA plate, incubated for 4 days, and harvested. Chromosomal DNA was prepared using a CTAB/Chloroform/Phenol extraction, as described [30]. In transformations utilizing donor PCR products, 9 kb PCR amplicons were first generated using primers flanking the Str^R^ mutation (A128G) and a mutation conferring rifampin resistance (G1588A in codon 530 of *rpoB*, resulting in D530V). Cells of strain 26695 were exposed to either 100 ng of PCR products containing both mutations or an equimolar mixture of two different amplicons (100 ng each), each containing only one mutation. Serial dilutions were inoculated to TSA plates, 20 µg/ml streptomycin plates, 87.5 µg/ml rifampin plates, and to plates containing both streptomycin and rifampin.

Each Cm^R^ transformant was picked with a sterile pipette, inoculated onto a separate BS agar plate with 10 µg/ml chloramphenicol, and incubated for 3 days. The culture from the selective plate was swabbed onto a TSA plate, incubated for 2 days, and harvested. Chromosomal DNA was prepared using the Wizard genomic DNA purification kit (Promega, Madison, WI). To analyze the region of DNA flanking the *cat* cassette, PCR was performed using chromosomal isolates from 10 independent transformants. Primers were designed to match the sequences in both 26695 and J99 (Table S2). The desired PCR products were purified with the QIAquick PCR purification kit (Qiagen, Valencia, CA).

### DNA Sequencing

For analysis of specific sequences flanking the Str^R^ mutation, 0.5–1 kb PCR products were prepared as described above and were sequenced on both strands at the DNA Sequencing Resource Center (Rockefeller University, New York, NY). For analysis of specific sequences flanking the *cat* cassette, ∼1 kb PCR products were prepared as described above and sequenced on both strands at the High-Throughput Genomics Unit (University of Washington, Seattle, WA). All sequence analysis was performed using Sequencher software (Gene Codes, Ann Arbor, MI).

### Statistical analysis of imports

If the distribution of imports is assumed to be exponential with mean length λ, and imports are randomly distributed on the chromosome, then the probability density function of imports of length *x* containing the Str^R^ mutation will be equal to 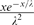, since the probability that any given import will include the Str^R^ site is proportional to its length. Not all nucleotides differentiate 26695 from J99, so the length of each observed import is ambiguous. If an import has possible start positions ranging from *a* to *b* and end positions from *c* to *d* then the likelihood of observing it is 
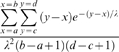
. The overall likelihood of the data given λ is the arithmetic product of the likelihoods of all imports.

Restriction modification could result in some sites being more likely to contain the endpoints of imports than others. We model this by assuming that unmethylated sites for which the restriction modification system is present in the recipient strain are γ times more likely to be digested than other sites. The likelihood of observing a particular event of length, starting at 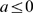 and finishing at site 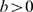, conditional on it containing the Str^R^ mutation at site 0 then is proportional to 
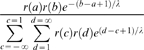
, where 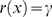 if *x* is at an unmethylated restriction site and 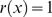 otherwise. This expression is approximated by truncating the sum at values of *c* and *d* that bound all observed imports (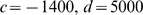 for imports from 26695 to J99 and 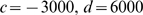 for imports from J99 to 26695).

### Genbank GeneID numbers

See Table 3 for a list of GenBank ID numbers.

**Table 3:** Genbank GeneID numbers.

Gene	Description	GeneID
rpsL	30S ribosomal protein S12 [*Helicobacter pylori 26695*]	899959
rpsL	30S ribosomal protein S12 [*Helicobacter pylori J99*]	889755
rpoB	DNA-directed RNA polymerase subunit beta/beta' [*Helicobacter pylori 26695*]	899960
rpoB	DNA-directed RNA polymerase subunit beta/beta' [*Helicobacter pylori J99*]	889766
recG	ATP-dependent DNA helicase [*Helicobacter pylori 26695*]	899768
recG	ATP-dependent DNA helicase [*Helicobacter pylori J99*]	889476

## Supporting Information

Table S1 Primers used to assess DNA crossover sites
(0.06 MB DOC)

Table S2 Primers for PCR *cat* cassette flanking region
(0.04 MB DOC)
