# Supplementary material for: Natural Transformation of Helicobacter pylori Involves the Integration of Short DNA Fragments Interrupted by Gaps of Variable Size
Source: PLoS Pathog. 2009 Mar 13;5(3):e1000337. doi: 10.1371/journal.ppat.1000337 (PMC2650093; doi:10.1371/journal.ppat.1000337)
Supplement: Table S1 — Primers used to assess DNA crossover sites (0.06 MB DOC) [file ppat.1000337.s001.doc]

**Table S1. Primers used to assess DNA crossover sites.**

| **Primer Designation** | **Sequence (5’-3’)** | **Orien-tation** | **Genomic location in 26695 /**  **J99** | **Prod.size**  **(bp)** | **RE site in J99**  **(Distance to A263G)** | **RE site in 26695**  **(Distance to A128G)** |
| --- | --- | --- | --- | --- | --- | --- |
| FlankHaeIIIF | CGGGTTGTTTGAACATGA | R | 1266647-630 /  J1244997-980 | 645 | *XcmI*  (1620bp) | *HaeIII*  (1743bp) |
| FlankHaeIIIR | CGATGATTTCTAGGTGCA | F | 1266003-020 /  J1244353 -370 |
| FlankHgaIF | GCCAGCAGTCGCTATCTA | R | 1267586-569 /  J1245936-919 | 682 | ___ | *HgaI*  (834bp) |
| FlankHgaIR | GGCACAGGATTAGCTTTC | F | 1266905- 922 /  J1245255-272 |
| FlankHhaIF | GAGAAGAAGAAAAGCACC | R | 1267863-846 /  J1246213-196 | 595 | *HinP1I*  (749bp) | *HhaI*  (520bp) |
| FlankHhaIR | TCTTATGGCTCACGCCTG | F | 1267269-286 /  J1245619-636 |
| Flank172HhaIF | CGCTTGAGGATAACTCTA | R | 1268401-384 /  J1246751-734 | 592 | ___ | *HhaI*  (172bp) |
| Flank172HhaIR | TTCCCATAAACAGGATCG | F | 1267810-827 /  J1246159-176 |
| Flank110AluIF | CAAGTTTATTGAAGGGGA | R | 1268704-687 /  J1247053-036 | 635 | *MboII*  (324bp) | *AluI*  (110bp) |
| Flank110AluIR | GTTATGCCCTTCACCAGG | F | 1268070-087 /  J1246419-436 |
| FlankDdeIF | GCGGGGATAGCAAGTTTA | R | 1268714-697 /  J1247063-046 | 504 | ___ | *DdeI*  (399bp) |
| FlankDdeIR | TTCCACTAATGCAGGTGA | F | 1268211-228 /  J1246560-577 |
| 399-1450F | ATCGCTAGGGGTTCTGAG- _TTC | R | 1269525-505 /  J1247874-854 | 723 | *ApoI*  (1214bp) | *HaeIII,,*  *DdeI*  (811, 1148) |
| 399-1450R | TTGCTGGACTTCGCTCAC | F | 1268803-820 /  J1247152-169 |
| FlankAlwIF | CTTCTATTTTAGTGGTAG- _AGCCTAA | R | 1270013-989 /  J1248362-338 | 586 | *HinfI*  (1450bp) | *AlwI*  (1450bp) |
| FlankAlwIR | GCAATCGTTTTGAAAGTG | F | 1269428-445 /  J1247777-794 |
| FlankHpaIIF | CAAATCCGTCAGCTTTCA | R | 1270749-732 /  J1249098-081 | 521 | ___ | *HpaI*  (2170bp) |
| FlankHpaIIR | CTCATCTTGCCTTCG | F | 1270229-243 /  J1248578-592 |
| Flank+3331 HaeIIIF | AAACAGGCTAAACGCATG | R | 1271700-683 /  J1250049-032 | 600 | ___ | *HaeIII*  (3331bp) |
| Flank+3331 HaeIIIR | ATAATCCACAAGCACGCC | F | 1271101-118 /  J1249450-467 |
| Flank+HhaIF | GTTGTATTATGAAGCTTA | R | 1272496-479 /  J1250845-828 | 373 | ___ | *HhaI*  (4191bp) |
| Flank+HhaIR | GACTAAAGGCCTTAAATC | F | 1272124-141 /  J1250473-490 |
