# Supplementary material for: Natural Transformation of Helicobacter pylori Involves the Integration of Short DNA Fragments Interrupted by Gaps of Variable Size
Source: PLoS Pathog. 2009 Mar 13;5(3):e1000337. doi: 10.1371/journal.ppat.1000337 (PMC2650093; doi:10.1371/journal.ppat.1000337)
Supplement: Table S2 — Primers for PCR cat cassette flanking region (0.04 MB DOC) [file ppat.1000337.s002.doc]

**Table S2. Primers for PCR *cat* cassette flanking region.**

| **Primer** | **Sequence (5’-3’)** | **Genomic location in 26695 /**  **J99** |
| --- | --- | --- |
| recGCmdownstream1F | GGCTAAGTCAATGTATTCAAAACT | 1602169-1602192/J1552347-1552370 |
| recGCmdownstream1R* | GGGCGTATTGCCAAAATAGTGGTC |  |
| recGCmdownstream2F | TTTCATCGCATAAAAGTTTGAGTTG | 1601420-1601444/J1551596-1551620 |
| recGCmdownstream2R | CACGATCCAAGAAGAAAACGAACG | 1602322-1602299/J1552500-1552477 |
| recGCmdownstream3F | CCTTTCTTCGGTGATGTCAAAAATG | 1600543-1600567/J1550729-1550753 |
| recGCmdownstream3R | CAAGAACCTTTTTGGGTCAAAATGC | 1601574-1601550/J1551750-1551726 |
| recGCmdownstream4F | CCCTAATTTAATGGCGTTGCTTGG | 1599743-1599766/J1549929-1549952 |
| recGCmdownstream4R | AAGAGCGATTATCAAAAATTAAGTG | 1600739-1600715/J1550918-1550894 |
| recGCmdownstream5F | TCGCTTAAATAGCTATTCACCCCG | 1598931-1598954/J1549111-1549134 |
| recGCmdownstream5R | CAAAAAATAACCCAGGGGAGCGTGG | 1599916-1599892/J1550102-1550078 |
| recGCmupstream1F* | GACTATCTACTGCCGATATTTACGT |  |
| recGCmupstream1R | GAGCAAAGCTCTTTTAATCAAGCT | 1603662-1603631/J1553840-1553817 |
| recGCmupstream2F | TCTTTAACGCCTTCCTTTTTTAA | 1603496-1603518/J1553674-1553696 |
| recGCmupstream2R | CCACCTGGTATTGAGAAGCCACATA | 1604515-1604491/J1554688-1554664 |
| recGCmupstream3F | CTCAACAGGAAGTATTAAAGTGTG | 1604371-1604394/J1554544-1554567 |
| recGCmupstream3R | CGGTCATTGTGTGTGGGGATTTGA | 1605364-1605341/J1555537-1555514 |
| recGCmupstream4F | GGGTAAAAATAACGGAAAGTGTCA | 1605205-1605228/J1555378-1555401 |
| recGCmupstream4R | CCAGCGAATTGGCTTATTCTTCCACAC | 1606251-1606225/J1556424-1556398 |
| recGCmupstream5F | GAGAGTTTTCAATACTCAAAGGCG | 1606003-1606026/J1556176-1556199 |
| recGCmupstream5R | AACGCGCCCATGCAAAAACCACAAA | 1607038-1607014/J1557211-1557187 |

* *Cat* cassette-specific primers
